# Supplementary figures and images for: Met32 governs transcriptional control of sulfur metabolic flexibility and resistance to reactive sulfur species in the human fungal pathogen Candida albicans
Source: mBio. 2026 Apr 20;17(5):e00472-26. doi: 10.1128/mbio.00472-26 (PMC13170222; doi:10.1128/mbio.00472-26)

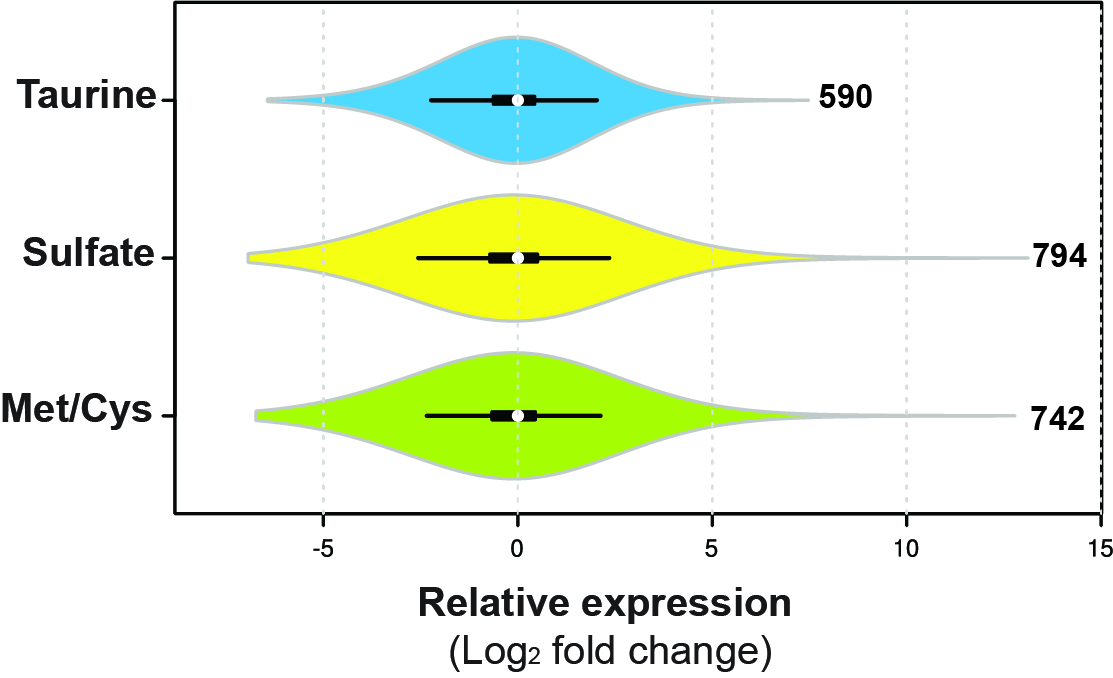

Supplement: Figure S1 — Violin plots showing the distributions of average relative expression levels across the different RNA-seq comparisons. [file mbio.00472-26-s0001.tif]

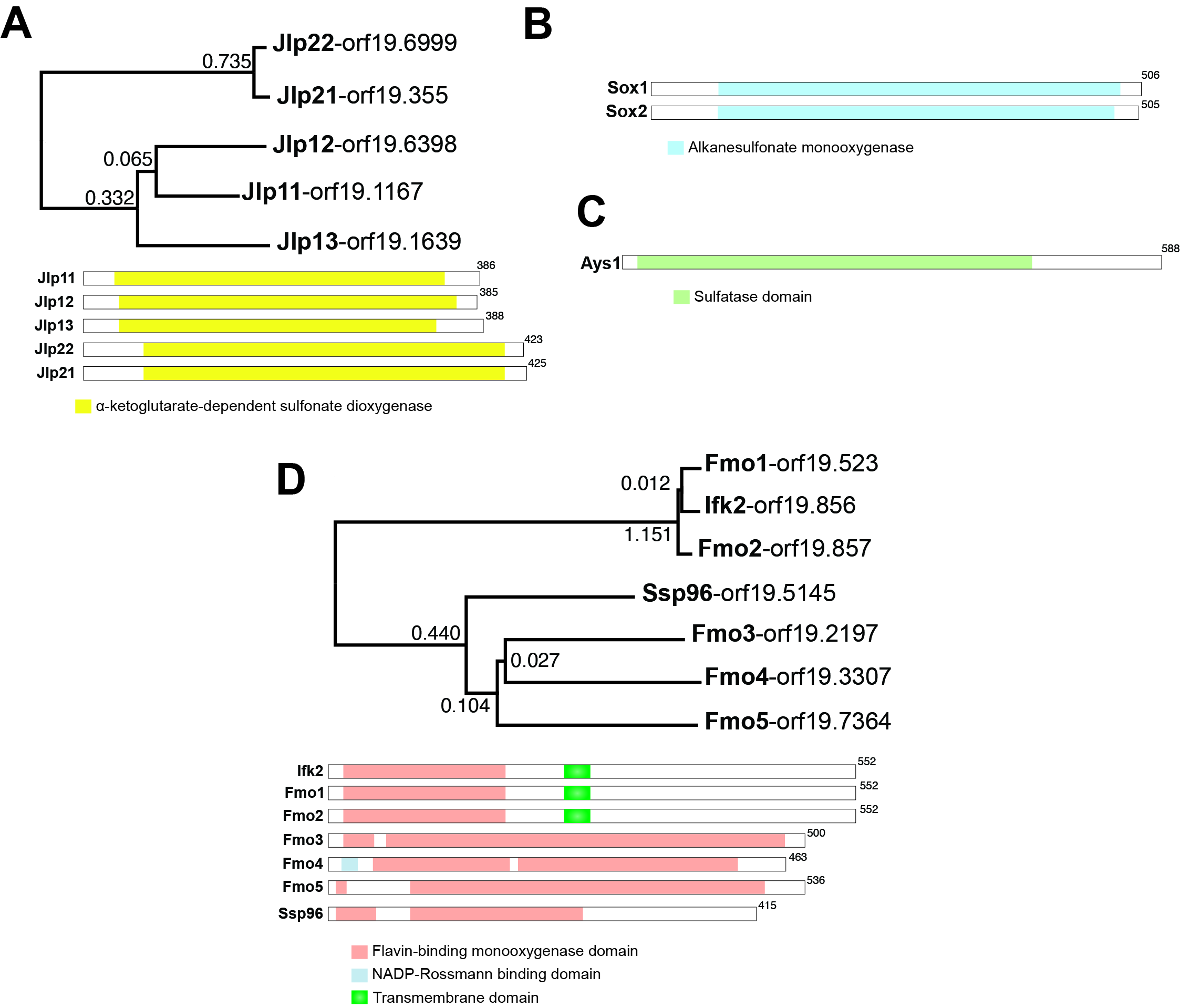

Supplement: Figure S2 — Phylograms and functional domain organization of C. albicans desulfonation proteins. [file mbio.00472-26-s0002.tif]

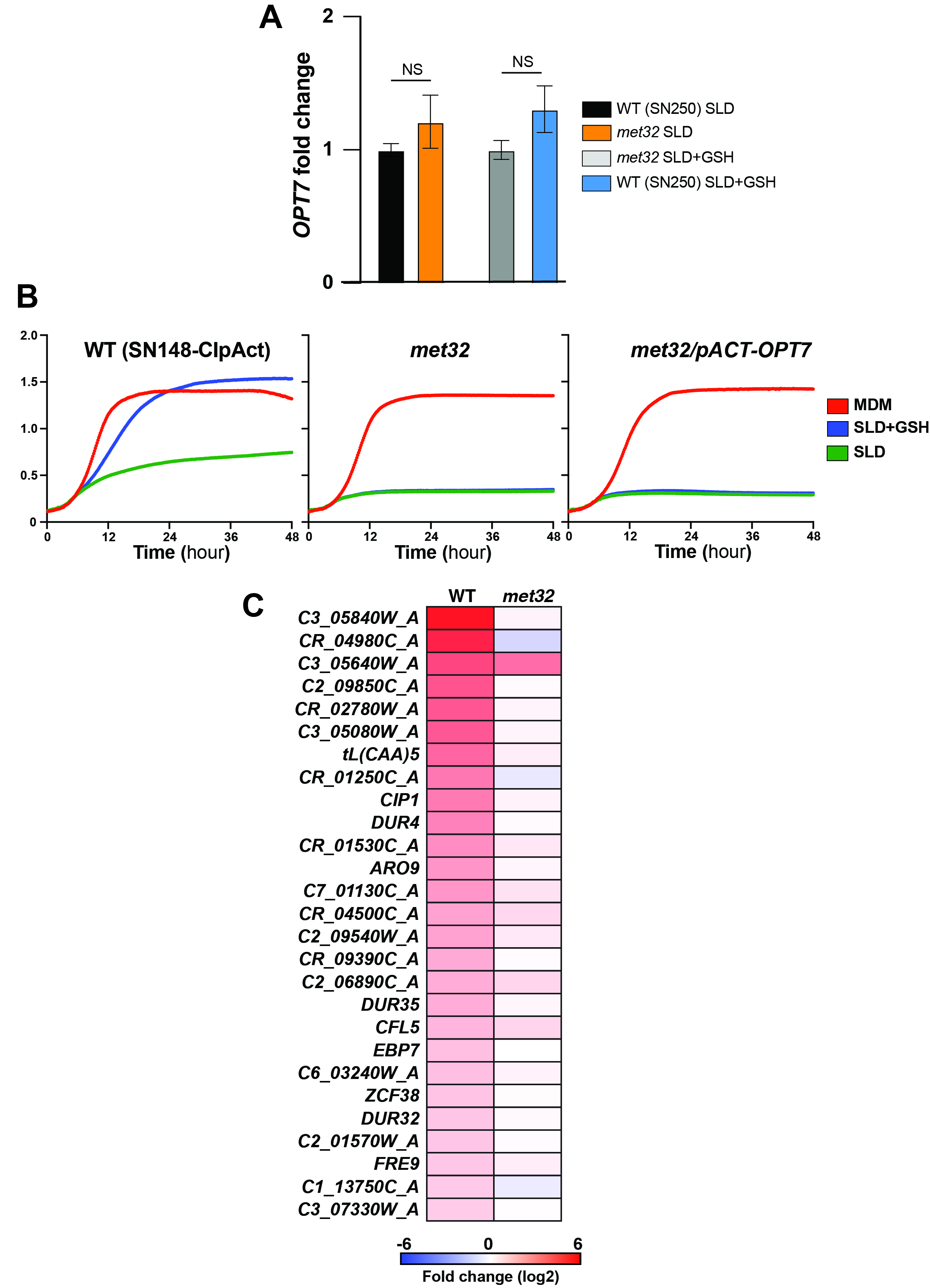

Supplement: Figure S3 — Transcript levels of the C. albicans GSH transporter OPT7 are not altered in met32 mutant. [file mbio.00472-26-s0003.tif]

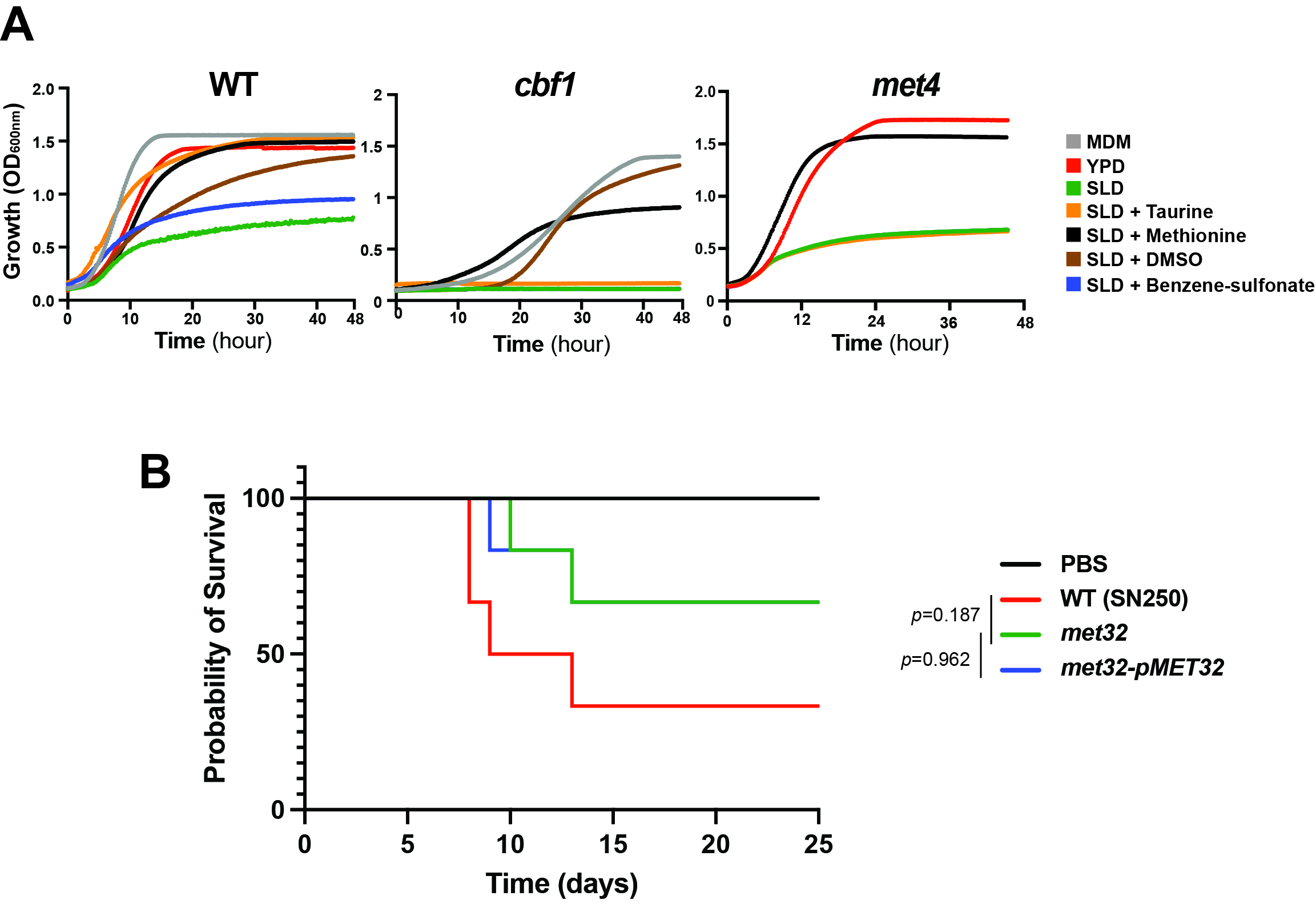

Supplement: Figure S4 — Cbf1 and Met4 requirement for sulfur utilization in C. albicans and survival curves of mice following infection with C. albicans strains. [file mbio.00472-26-s0004.tif]
