# Supplementary material for: Met32 governs transcriptional control of sulfur metabolic flexibility and resistance to reactive sulfur species in the human fungal pathogen Candida albicans
Source: mBio. 2026 Apr 20;17(5):e00472-26. doi: 10.1128/mbio.00472-26 (PMC13170222; doi:10.1128/mbio.00472-26)
Supplement: Legends — for the supplemental figures. [file mbio.00472-26-s0005.docx]

**Supplementary Figures**

**Figure S1.** Violin plots showing the distributions of average relative expression levels across the different RNA-seq comparisons using the three sulfur-replete source conditions: mixture of methionine and cysteine (Met/Cys), ammonium sulfate and taurine. The number of differentially modulated transcripts are indicated for each experiment.

**Figure S2**. Phylograms and functional domain organization of *C. albicans* desulfonation proteins including five sulfonate/α-ketoglutarate dioxygenases (**A**), two alkanesulfonate monooxygenases (**B**), the arylsulfatase Ays1 (**C**), and seven FMNH_2_-dependent monooxygenases (**D**)**.**

**Figure S3.** (**A**) Transcript levels of the *C. albicans* GSH transporter *OPT7* are not altered in *met32* mutant. Transcript levels of *OPT7* were measured by qPCR and fold-changes were calculated using the comparative Ct method. For each strain, data were normalized relative to the SLD condition. (**B**) Increasing *OPT7* dosage does not rescue the GSH utilization defect of the *met32* mutant. WT (WT-pAct), *met32* and *met32* overexpressing *OPT7* (*met32*- pAct-*OPT7*) were grown in SLD with 80 µM GSH at 30°C. OD_600_ readings were taken every 10 min for 48 hours. (**C**) Heatmap representation of Met32-modulated transcripts related to different biological processes other than sulfur metabolism. Heatmap showing the modulation of sulfur-related transcripts is indicated in **Figure 5D**.

**Figure S4.** (**A**) The transcription factors Cbf1 and Met4 are required for sulfur utilization in *C. albicans*. WT (CAI4), *cbf1* and *met4* strains were grown in MDM, YPD, SLD alone or supplemented with 80 µM of different sulfur sources (taurine, methionine, benzene sulfonate and dimethyl sulfoxide (DMSO). Cells were grown at 30°C, and OD_600_ readings were taken every 10 min for 48 hours. (**B**) Survival curves of mice following the infection with *C. albicans* WT (SN250), the *met32* mutant and the *met32*-p*MET32* complemented strain. Mice with cyclophosphamide-induced immunosuppression were injected with phosphate-buffered saline (PBS) or 7 x 10^3^ CFU of each *C. albicans* strain. Survival differences were evaluated using Kaplan–Meier survival analysis with log-rank (Mantel-Cox) test with correction for multiple comparisons.
